# *FGFR2*-fusions define a clinically actionable molecular subset of pancreatic cancer

**DOI:** 10.1038/s41698-024-00683-x

**Published:** 2024-09-17

**Authors:** Leah Stein, Karthikeyan Murugesan, Julie W. Reeser, Zachary Risch, Michele R. Wing, Anoosha Paruchuri, Eric Samorodnitsky, Emily L. Hoskins, Thuy Dao, Amy Smith, Dat Le, Melissa A. Babcook, Yi Seok Chang, Matthew R. Avenarius, Muhammad Imam, Aharon G. Freud, Sameek Roychowdhury

**Affiliations:** 1 Comprehensive Cancer Center and James Cancer Hospital, The Ohio State University, Columbus, OH USA; 2 Biomedical Sciences Graduate Program, The Ohio State University, Columbus, OH USA; 3 Foundation Medicine Inc, Cambridge, MA USA; 4 Department of Pathology, The Ohio State University, Columbus, OH USA; 5 Florida Cancer Specialists, Orlando, FL USA; 6 Division of Medical Oncology, Department of Internal Medicine, The Ohio State University, Columbus, OH USA

**Keywords:** Cancer genomics, Oncogenes, Pancreatic cancer, Predictive markers, Translational research

## Abstract

Genomic alterations in fibroblast growth factor receptor (*FGFR)* genes are present in a small number of metastatic pancreatic ductal adenocarcinomas (PDAC) and may represent an emerging subgroup of patients likely to benefit from FGFR targeted therapies. Here we present four *FGFR2* fusion-positive metastatic PDAC patients who exhibited durable responses or disease control to FGFR kinase inhibitors. Utilizing our custom *FGFR* focused cell-free DNA assay, FGFR-Dx, we serially monitored variant allele fractions of *FGFR2* fusions during FGFR inhibitor treatment and observed dynamic changes correlating with clinical responses. Genomic analysis of 30,229 comprehensively profiled pancreatic cancers revealed *FGFR1-3* fusions in 245 cases, an incidence of 0.81%. *FGFR* fusions were generally mutually exclusive from other known oncogenes. Our findings provide clinical evidence for identifying and treating *FGFR2* fusion-positive PDAC patients with FGFR targeted therapy.

## Introduction

Pancreatic ductal adenocarcinoma (PDAC) is the fourth leading cause of cancer-related death in the United States and Europe, with the median overall survival for advanced disease being less than 12 months^1–4^. PDAC is predominantly characterized by *KRAS* mutations, which are found in approximately 90% of patients. The remaining 10% have a variety of other rare oncogenic driver alterations^5,6^. Recent retrospective studies in advanced pancreatic cancer have suggested that biomarker-selected patients receiving molecularly targeted therapy have improved overall survival^7^.

**Fig. 1:**
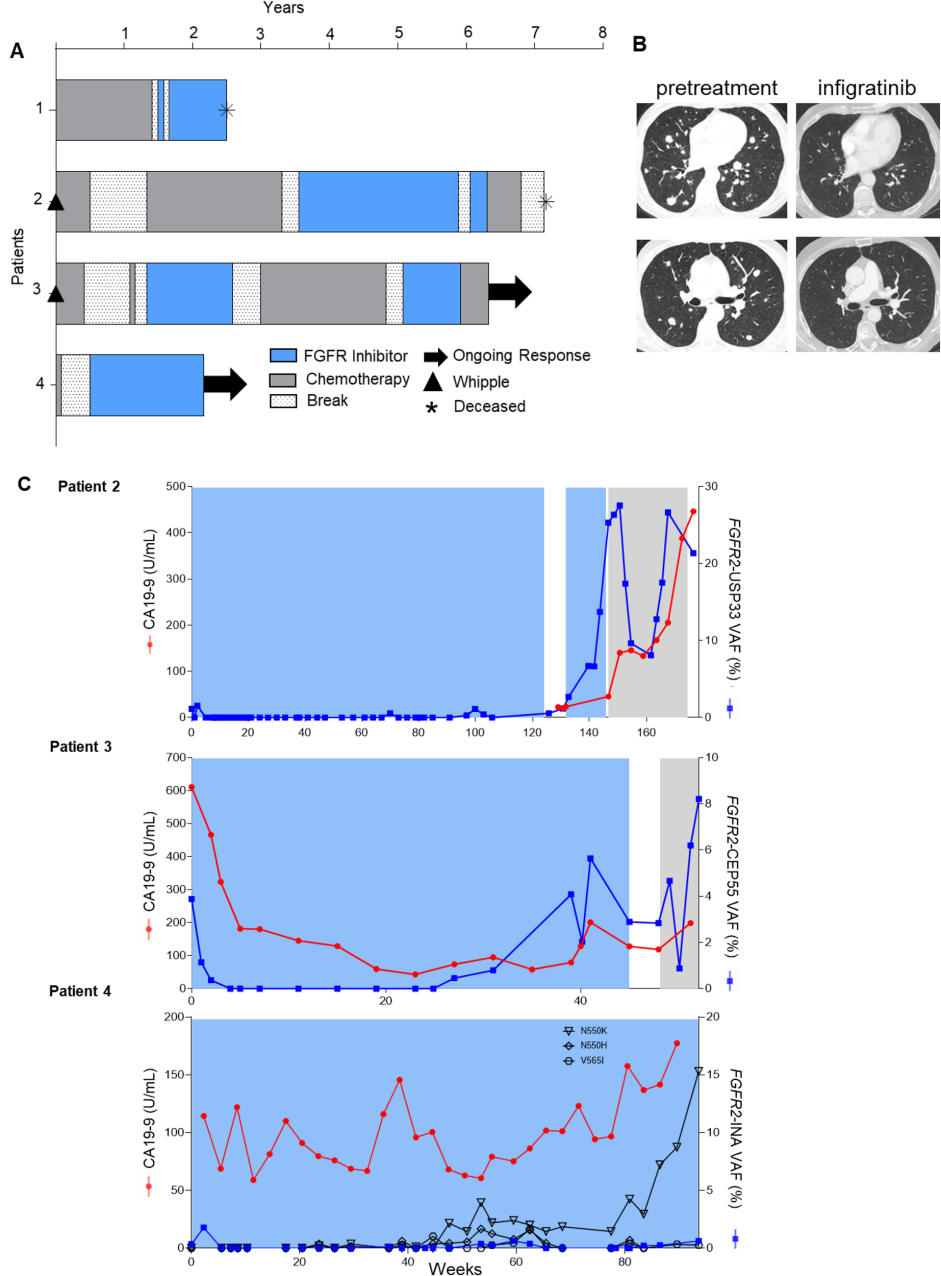
Patients with metastatic pancreatic cancer and *FGFR2* fusions demonstrate response and clinical benefit when treated with FGFR kinase inhibitors. **A** Overview of treatment time course in four patients with metastatic pancreatic cancer who received FGFR kinase inhibitors. Patient 1 had stable disease as best response, while patient 2-4 experienced partial responses. **B** Partial response to FGFR kinase inhibitor infigratinib after 10 months of treatment in patient 3 demonstrated by decreased size of lung metastases. Left is pretreatment CT scans and right is after 10 months on infigratinib. **C** Serial monitoring of CA19-9 and plasma cell free DNA for *FGFR2* fusion during FGFR inhibitor targeted therapy. Patient 4 developed known *FGFR2* resistance mutations alongside of his fusion VAF (%). Areas shaded in blue represent FGFR inhibitor treatment, areas shaded in gray represent chemotherapy treatment, and non-shaded areas represent no treatment. VAF, Variant Allele Frequency.

Tumor agnostic basket trials have helped identify new subsets of patients who may benefit from targeted therapies. Since 2014, three *FGFR2* fusion-positive metastatic PDAC patients participated in FGFR inhibitor basket trials at Ohio State University. One additional patient received an FGFR inhibitor off-label. Three of the four patients participated in a serial liquid biopsy study (NCT02090530). All four patients had canonical fusion breakpoints involving intron 17 and exhibited durable responses or disease control to FGFR inhibitors with survival ranging from two to seven years (Fig. 1A–C, Supplementary Figs. 1–2). Here we present clinical histories and blood-based biomarker trends for the carbohydrate antigen 19-9 (CA19-9) tumor marker and *FGFR2* fusion level measured by FGFR-Dx, a novel *FGFR*-focused sequencing assay (Fig. 1C). Further, we provide a comprehensive landscape of clinically actionable *FGFR1-4* fusions across 30,229 pancreatic cancers.

## Results

### Clinical course of patients with FGFR fusion-positive metastatic pancreatic cancer

Patient 1 was a 60-year-old man who first received gemcitabine and nab-paclitaxel for four months followed by folinic acid, fluorouracil, and oxaliplatin (FOLFOX) for 12 months (Fig. 1A, Supplementary Figs. 1–2A, Supplementary Table 1). After the detection of an *FGFR2-INA* fusion, he enrolled in a basket trial for ponatinib (NCT02272998), a multi-targeted tyrosine kinase inhibitor (multi-TKI) re-purposed for *FGFR* alterations (Supplementary Fig. 3). After only two weeks, he developed venous thrombosis and ponatinib was therefore discontinued per study rules. He then received another off-label multi-TKI, pazopanib, and tolerated this well with no side effects. He exhibited stable disease for 10 months before passing due to disease progression (Fig. 1A).

Patient 2 was a 44-year-old woman who underwent a Whipple surgery followed by six months of gemcitabine and cisplatin (Fig. 1A, Supplementary Fig. 1, Supplementary Table 1). After 10 months of surveillance, metastatic disease was detected in the right hepatic lobe. She then received gemcitabine, nab-paclitaxel, and the STAT3 inhibitor BBI608 (Napabucasin) for 10 months followed by folinic acid, fluorouracil, and irinotecan (FOLFIRI) and BBI608 for 14 months through a clinical trial at the Ohio State University where she experienced a partial response (NCT02231723). Following disease progression on this trial, genomic testing revealed an *FGFR2-USP33* fusion, and she subsequently started a basket trial for the FGFR-selective inhibitor pemigatinib where she exhibited a partial response with a 43% reduction in tumor size (NCT02393248) and remained on therapy for 28 months (Fig. 1A, Supplementary Figs. 1, 2B, 4)^8^. After imaging showed worsening metastatic lymphadenopathy, she enrolled in a second basket trial with the FGFR*-*selective TKI infigratinib (NCT04233567) where she exhibited stable disease after nine weeks (Supplementary Figs. 2B, 5, 6). Her next scans showed progressive disease and her treatment was subsequently switched to FOLFOX. Unfortunately, her cancer did not respond to FOLFOX and she passed away due to growing disease burden (Fig. 1A, Supplementary Fig. 1).

Patient 3 is a 69-year-old man who underwent a Whipple surgery and received adjuvant gemcitabine and capecitabine for 14 months. During surveillance, positron emission tomography/computerized tomography (PET/CT) revealed a hypermetabolic lesion in the right hepatic lobe (Fig. 1A, Supplementary Fig. 1). He briefly started gemcitabine and nab-paclitaxel but stopped due to side effects. Following the detection of an *FGFR2-CEP55* fusion, he was offered a ponatinib basket trial (NCT02272998) and experienced stable disease with 21.2% reduction in target lesion size for 15 months (Fig. 1A, Supplementary Fig. 1, Supplementary Fig. 3, Supplementary Fig. 7). Due to peritoneal disease progression and development of lung nodules, he changed to gemcitabine-cisplatin/carboplatin for 16 months with stable disease (Fig. 1A, Supplementary Fig. 1). Again, due to progression, he was offered an infigratinib basket trial (NCT04233567) and exhibited a partial response including a 40% reduction in target lesions for 10 months (Fig. 1A–C, Supplementary Fig. 5, Supplementary Fig. 8). Due to persistent grade 3 corneal ulceration, infigratinib was discontinued per study rules and gemcitabine plus carboplatin was resumed.

**Fig. 2:**
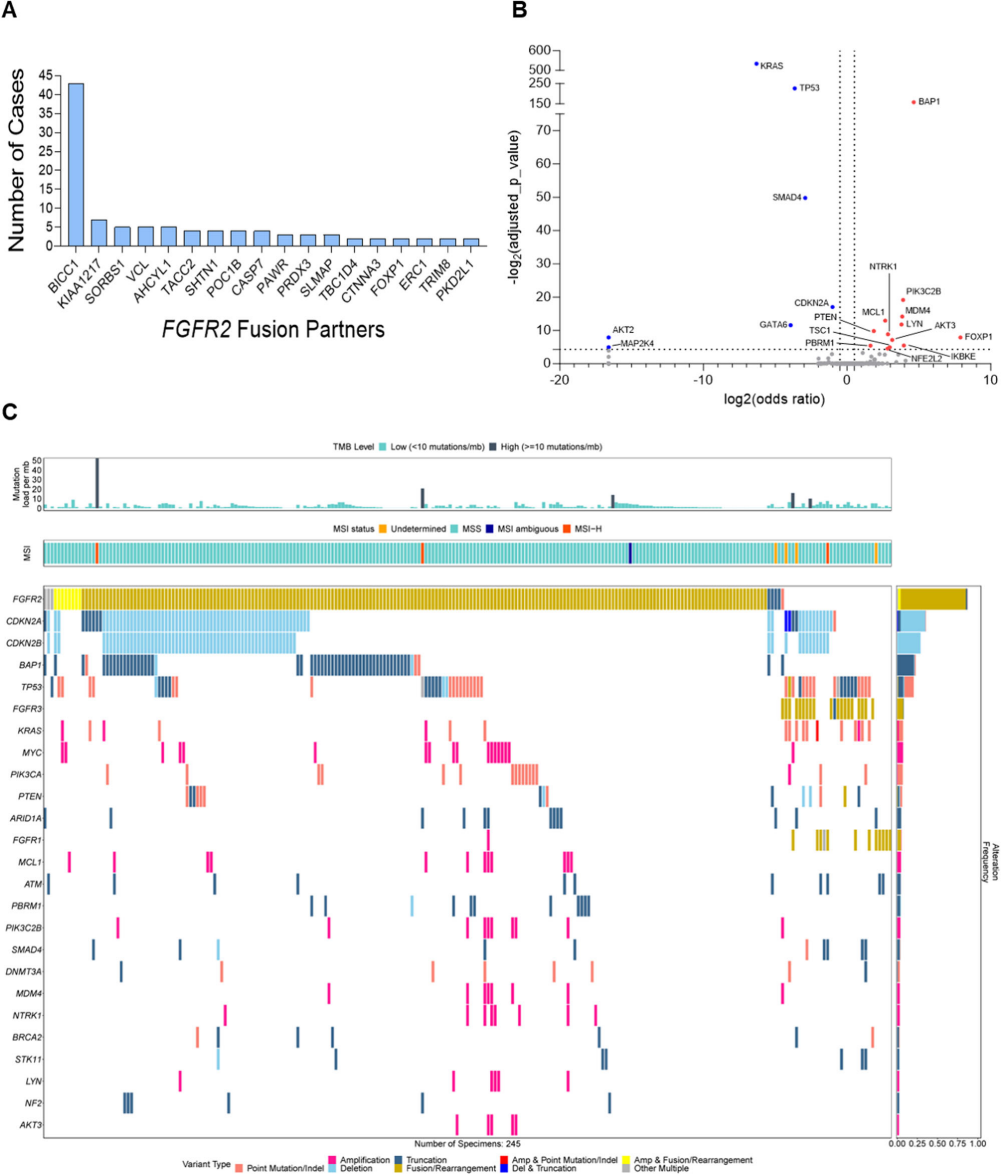
Genomic landscape of *FGFR2* fusion-positive pancreatic cancer. **A** The most common *FGFR2* fusion partners found in pancreatic cancer (*N* = 30,229). **B** Volcano plot of the *FGFR2* fusion cohort of co-occurring (red dots) and mutually exclusive (blue dots) with the *FGFR* alteration, respectively. Only genes with an adjusted *p* ≤ 0.05 and gene prevalence ≥0.5% were labeled. **C** Oncoplot of the *FGFR2* fusion cohort illustrating co-occurring alterations, tumor-mutational burden (TMB), and MSI status.

Patient 4 is a 78-year-old man initially treated with gemcitabine and nab-paclitaxel but discontinued after one month due to acute kidney injury, dehydration, and diarrhea requiring hospitalization (Supplementary Table 1). A biopsy revealed an *FGFR2-INA* fusion, and he was offered off-label pemigatinib and experienced a decrease in tumor size with ongoing disease control for 20 months (Figs. 1A, C, 2C).

### cfDNA reveals correlation between fusion VAF (%) and treatment response

Two blood-based biomarkers were examined for correlation with treatment response in three of the previously described patients. The first biomarker was carbohydrate antigen 19-9 (CA19-9), which is elevated in the blood of most patients with pancreatic cancer^9^. Second, we applied an *FGFR* targeted sequencing assay, FGFR-Dx, to serially monitor the variant allele fraction (VAF) of *FGFR2* fusions in plasma cfDNA from patients 2, 3, and 4 throughout treatment. Patient 2’s CA19-9 was not abnormal prior to starting pemigatinib (18.0 U/mL) or infigratinib (20.2 U/mL). Therefore, CA19-9 was not monitored during either FGFR inhibitor treatment. Before starting pemigatinib, her plasma cfDNA *FGFR2-USP33* fusion was detected at 1.1% VAF and subsequently decreased to an undetectable level two weeks after starting treatment (Fig. 1C). The fusion remained undetectable until time of progression at 100 weeks with the exception of one sample at 70 weeks (1.12%). Upon starting infigratinib, the fusion VAF was 2.7% but rose to 13.7% over 14 weeks corresponding with disease progression on CT and MRI imaging. Her fusion VAF then rapidly increased to 26.4% before resuming FOLFOX. In response to FOLFOX, the VAF decreased to 8.1%, but further increased to 26.7% just prior to passing, which correlated with a rapid rise in CA19-9 (Fig. 1C). A similar trend was observed in patient 3. His *FGFR2-CEP55* fusion VAF was 3.9% when starting infigratinib, became undetectable after 4 weeks, remained undetectable for 23 weeks, and then slowly increased to 2.9% when treatment was stopped due to the emergence of corneal ulcers. Concurrent testing with Tempus xF after 50 weeks on infigratinib failed to detect his *FGFR2-CEP55* fusion, however, FGFR-Dx detected it at 2.84%^10^. Upon resuming gemcitabine plus carboplatin, his fusion VAF briefly decreased and then rapidly increased to 8.2%. Overall, the response of the fusion VAF correlated with both CA19-9 and imaging (Fig. 1B, C). Patient 4’s *FGFR2-INA* fusion VAF was also monitored consistently through pemigatinib treatment starting at 0.35% and fluctuated slightly throughout his 86 weeks of pemigatinib treatment, perhaps related to the two weeks on-therapy and 1 week off-therapy schedule. His *FGFR2-INA* fusion VAF remains below 0.65% at the time of publication (Fig. 1C). Similar to patient 3, concurrent testing with Guardant 360 failed to detect his *FGFR2-INA* fusion while FGFR-Dx detected it at 0.62% after 90 weeks on pemigatinib^11,12^. In contrast, his CA19-9 levels were quite variable throughout treatment and did not seem to capture the clinical benefit apparent by measuring *FGFR* fusion levels in plasma cfDNA.

We also analyzed cfDNA samples for *FGFR* single nucleotide variants (SNVs), which has been shown to be an effective method of monitoring the emergence of acquired resistance to FGFR inhibitors in cholangiocarcinoma^13^. In patients 2 and 3, no high confidence SNVs were detected. In Patient 4, we detected *FGFR2 N550H*/*K* and *V565I* after 24 and 36 weeks of treatment, respectively. All three SNVs demonstrate some variance while on treatment (Fig. 1C). Notably, VAFs for FGFR2-INA and point mutations in patient 4 are different, since different tools are used to measure variant supporting reads. These resistance SNVs have been shown to confer resistance to FGFR inhibitors by reducing inhibitor binding affinity or causing autophosphorylation of the *FGFR2* tyrosine kinase domain^14–16^.

### Investigating the landscape of FGFR genomic alterations across pancreatic cancer

Based on the exceptional response of these patients, we investigated the landscape of *FGFR* genomic alterations across pancreatic cancers. We evaluated 30,229 tumors with a clinical diagnosis of pancreatic cancer tested with FoundationOne (F1) and F1CDX® assays (Foundation Medicine, Inc., Cambridge, MA, USA) (Supplementary Table 2). 6.9% (2,079/30,229) of patients harbored *FGFR1-3* alterations, including rearrangements (REs), copy number amplifications (CNAs), and short variants (SVs) (Supplementary Fig. 9). Of the *FGFR*-altered cohort, REs were most observed in *FGFR2* at 11.3% (234/2,079), followed by *FGFR1*, 5.2% (108/2,079), and FGFR3 2.0% (42/2,079) (Supplementary Fig. 9). However, there are still many *FGFR1-3* short variants that include in-frame insertions/deletions, point mutations, and frameshift mutations that have yet to be characterized in patients with PDAC (Supplementary Fig. 9B). As seen in cholangiocarcinoma, we observed diverse fusion partners in pancreatic cancer including 114 unique partner genes. Common *FGFR2* fusions included: *FGFR2-BICC1* (43), *FGFR2-KIAA1217* (7) *FGFR2-SORBS1* (5), and *FGFR2-AHCYL1* (5) (Fig. 2A). For *FGFR1*, the most observed RE was *FGFR1-TACC1* (3). In *FGFR3*, the most common RE was *FGFR3-TACC3* (14).

Next, we investigated 245 *FGFR1-3* fusion-positive PDAC cases to assess the most common co-mutations that might impact the biology and clinical course of these patients. The most prevalent co-occurring alterations were *BAP1* truncations and *CDKN2A/B* deletions (Fig. 2B, C). Most tumors were microsatellite stable (MSS) and displayed a low tumor mutation burden (TMB). Evaluation of mutual exclusivity showed that *FGFR2* fusions generally occur in isolation, supporting their role as driving events (Fig. 2C). A subset (9.2%) of tumors had co-mutations in *PIK3CA* or *KRAS* (Fig. 2B, C).

In addition to gene fusions, there were gain-of-function SVs and CNAs in *FGFR1*-*3*. In this cohort of pancreatic cancer there were 18 unique known gain-of-function SVs reported, including *FGFR2* F276C (10), *FGFR2* C382R (6), *FGFR1* N546K (3), *FGFR2* Y375C (3), and *FGFR2* H167_N173del (2) (Supplementary Fig. 10). In addition, there were 563 unique SVs of unknown significance (VUS) that warrant further characterization (Supplementary Fig. 11). Although CNAs occur in *FGFR1-3*, these have not been as clinically actionable in past basket trials.

## Discussion

Our clinical observations are consistent with eight patients in case reports or basket trials involving *FGFR2* fusion-positive PDAC patients demonstrating clinical responses to FGFR inhibitors^15,17–20^. Here, we demonstrate a detailed clinical course, longer follow up, and positive clinical correlation with cfDNA for *FGFR2* fusions. Based on these observations, a prospective study of pancreatic cancer patients with *FGFR* genomic alterations is warranted but remains a challenge. While known gain-of-function *FGFR* genomic alterations constitute 1–1.5% of PDAC (600–1200 patients per year in the U.S.), a traditional prospective clinical trial could have challenges for accrual. With the increasing use of telemedicine, there is an emerging push to adapt towards decentralized clinical trials monitored virtually regardless of location^21^. Therefore, we are designing and implementing a telemedicine clinical trial for *FGFR*-altered pancreatic cancers to enable patient access and improve accrual for this ultra-rare precision oncology target (Supplementary Fig. 12). We have treated patient 4 with an FGFR inhibitor (pemigatinib) through telemedicine, further supporting the feasibility and safety of this approach.

In addition to positive outcomes of *FGFR2*-fusion positive PDAC patients, there are other studies outlining the clinical relevance of PDAC patients who have *KRAS* WT tumors. In a study investigating a cohort of 2483 PDAC patients, 266 (10.7%) patients were *KRAS* WT. This cohort exhibited a small, but significant prolongation of overall survival compared to *KRAS* mutant PDAC patients. Interestingly, 14 of these patients harbored *FGFR2* fusion and 5 harbored *FGFR3* amplifications^5^. More broadly, another study highlighted that *KRAS* WT PDAC patients who harbored clinically actionable targets and received a matched targeted therapy tended to have longer overall survival compared to patients who did not receive a matched targeted therapy^7^. Both these studies provide evidence for the potential of *FGFR* alterations as both predictive and prognostic biomarkers for PDAC patients.

This is the largest study thus far to investigate the prevalence *FGFR2*-fusions and other alterations in *FGFR* in PDAC while also highlighting the role these alterations have as predictive biomarkers for FGFR targeted therapy. A limitation of the study is that it is a case-level report of a small sample size of four patients. However, their clinical data provides strong support that *FGFR* alterations are clinical actionable in PDAC. Another limitation includes the use of non-specific FGFR inhibitors, ponatinib and pazopanib, in two of the four *FGFR2*-fusion positive patients. Patients 1 and 3 were treated with ponatinib and/or pazopanib due to lack of availability of more specific FGFR targeted therapies. While the FoundationOne cohort illustrates the prevalence of *FGFR* alterations in PDAC, this is limited by lack of further clinical annotation. As more patients with PDAC receive FGFR inhibitors, there will be further opportunities to understand the mechanisms of acquired resistance. Based on patient 4, where we observed resistant mutations seen in cholangiocarcinoma, future research and clinical trials will provide an opportunity to understand emerging resistance in PDAC.

In summary, there is a small subset, 1–1.5%, of metastatic PDAC patients with *FGFR* activating SVs and fusions who may clinically benefit from FGFR inhibitors with durable responses. We described four *FGFR2* fusion-positive PDAC patients who exhibited excellent clinical outcomes with FGFR inhibitors and illustrated the landscape of *FGFR* fusions and SVs in a large cohort of pancreatic cancers. These findings support the further study of *FGFR* genomic alterations as clinically actionable targets in PDAC.

## Methods

### Clinical samples

Patients were consented to genomic testing and review of their electronic medical records under the institutional review board-approved study OSU-13053, *Precision Cancer Medicine for Advanced Cancer through High-throughput Sequencing*. This study allows for serial evaluation of blood and tissue specimens for genomic analyses as well as lifetime follow-up to examine clinical outcomes (NCT02090530). In addition, patients 1–4 consent to our study OSU-13053 includes permission to publish their clinical and research findings.

### Blood collection and cfDNA isolation

Whole blood was collected in either EDTA (K2) tubes or Cell-Free DNA BCT® (Streck) and plasma was isolated after centrifugation for 10 min at 1000 rpm. Prior to cfDNA isolation using QIAamp Circulating Nucleic Acid Kit (Qiagen) per manufacturer’s recommendations, plasma samples were subjected to a second centrifugation for 10 min at full speed (14,000 rpm).

### Clinical tumor genomic testing for FGFR

Targeted RNAseq was performed for patients 1–3 as previously described (OSU-SpARKFuse)^22^. Patient 4 had genomic testing completed by FoundationOne®CDx (see detailed methods below).

### Sequencing and bioinformatics analysis of cfDNA (FGFR-Dx)

For the cfDNA samples, a novel *FGFR* targeted DNA-based sequencing assay, FGFR-Dx, was used to measure the variant allele frequencies of FGFR fusions and resistance SNVs. Biotinylated 120-mer probes (Integrated DNA Technologies, IDT) were designed to target all exons and selected introns of *FGFR1, 2*, and *3*. Because some of the targeted regions had very low sequence complexity or specificity, assay probes were split into two pools: low risk and high risk. For each patient’s initial cfDNA sample, both low risk and high risk probes were used to maximize fusion detection. Once the genomic breakpoint for the fusion was identified, high risk probes were included only if the breakpoint was found to be in a high risk region. 10–20 ng of cfDNA was used as input for library preparation using the KAPA Hyper Library Prep Kit (Roche). The same amount of genomic DNA from whole blood was used as input as a normal control for variant calling. 350–500 ng of each cfDNA or blood gDNA library were pooled (6 µg total input) and subjected to hybridization and capture with FGFR-Dx probes as previously described^22^. Sequencing was performed on an Illumina NovaSeq 6000 instrument. UMI-aware demultiplexing from raw BCL/CBCL files was performed with Illumina’s software bcl2fastq version v2.20.0.422, running on Java version 1.8.0. Initial alignment to hg19 was performed with BWA version 0.17.17 using the “mem” algorithm, GATK version 4.0.10, and SAMtools v1.16.1^23–25^. Consensus calling was performed using fgbio tools version 2.1.0 (https://github.com/fulcrumgenomics/fgbio), requiring at least 3 reads per family for SNVs and 1 read per family for fusions, composite bases with quality <30 masked, 5% maximum no-calls, and maximum per-base error rate 0.1. Consensus reads were re-aligned with BWA mem. This yielded ~3000x average post-consensus coverage. Single nucleotide variants (SNVs) were called utilizing VarDict version 1.8.2, with 0.1% minimum variant fraction, minimum base quality of 20, and minimum mapping quality of 10^26^. To qualify as a high confidence variant, we required a variant fraction of ≥1%, and a minimum of five variant supporting reads in each direction in at least one sample for a particular patient. All variant frequencies for high confidence variants were included in graphs for serial monitoring (Fig. 1). We did detect known resistance SNVs (N550H, N550K, L618V) in Patient 3. However, they did not meet our criteria as high confidence variants and were therefore not including in the serial monitoring graphs (Fig. 1). Each patient’s fusion was identified from tumor tissue biopsy and known prior to FGFR-Dx analysis. Fusions were called with Manta version 1.4.0 and fusion supporting reads were combined if multiple breakpoints were called within 150 bases of each other^27^. Computations were performed on the Owens cluster at the Ohio Supercomputer (https://www.osc.edu/). All *FGFR2* SNVs were annotated as the NM_022970.3 *FGFR2* transcript. Sequencing data will be submitted to the Database of Genotypes and Phenotypes (dbGaP, https://www.ncbi.nlm.nih.gov/gap/).

### Comprehensive genomic profiling

Comprehensive genomic profiling (CGP) of tissue specimens from 30,229 routine clinical pancreatic cancer cases was performed using the FoundationOne (F1) and FoundationOne CDx assays (F1 CDx; Foundation Medicine, Inc., Cambridge, MA, USA) assays, as described previously, in a Clinical Laboratory Improvement Amendments (CLIA)-certified and College of American Pathologists (CAP)-accredited laboratory ^28,29^. The dataset is, henceforth, referred to as the FoundationCORE dataset.

All samples submitted for sequencing featured a minimum of 20% tumor cell nuclear area and yielded a minimum of 50 ng of extracted DNA. CGP was performed on hybrid-capture, adapter ligation-based libraries, to identify genomic alterations [base substitutions, small insertions, and deletions, copy number alterations and rearrangements] in coding exons (F1CDx: *n* = 309; F1: *n* = 395) and select introns of cancer-associated genes (F1CDx: *n* = 36; F1: *n* = 31), and TMB, MSI. TMB was calculated as the number of nondriver somatic coding mutations per megabase (mut/Mb) of genome sequenced; TMB-high was defined as ≥10 mut/Mb and TMB-low as <10 mut/Mb^30^. MSI status was determined by analyzing 114 intronic homopolymer repeat loci for length variability and MSI high was defined as described previously^31^.

All genomic alterations studied included only those described as functional or pathogenic in the scientific literature and seen in the Catalog Of Somatic Mutations In Cancer (COSMIC) repository, or those with a likely functional status (frameshift or truncation events in tumor suppressor genes)^32^. Variants of unknown significance (VUS) were not studied. Approval for this analysis, including a waiver of informed consent and a Health Insurance Portability and Accountability Act (HIPAA) waiver of authorization, was obtained from the Western Institutional Review Board (IRB; Protocol No. 20152817).

### Statistical analyses

For mutual exclusivity, all statistical analyses were performed using R software v4.2.0 (R Foundation for Statistical Computing, Vienna, Austria) and Python v2.7.16 (Python Software Foundation, Wilmington, DE, USA). Proportions of categorical variables were compared using the Fisher’s exact test. All *P* values were two-sided, and multiple hypothesis testing correction was performed using the Benjamini–Hochberg procedure to estimate the false discovery rate.

### Clinical trial Information

**OSU-14078: Phase II study of ponatinib for advanced cancers with genomic alterations in fibroblastic growth factor receptor (*FGFR*) and other genomic targets (*KIT***, ***PDGFRa***, ***RET FLT3***, ***ABL1*) (NCT 02272998)**.

**Description:** This open-label, single arm phase 2 basket trial was a single institution study completed at The Ohio State University James Cancer Center in Columbus, Ohio, USA. Eligible patients included the following: aged 18 years or older, histologically or cytologically confirmed diagnosis of refractory metastatic solid tumor for whom no other standard treatment options are available, and the presence of activating genomic alterations in *FGFR* (mutations, fusions or amplifications (>6 copies)) or activating genomic alterations in *KIT, PDGFRα, RET, ABL1* and *FLT3* by any validated CLIA-certified molecular testing (FISH, PCR or sequencing data were acceptable). This study included two patients with pancreatic cancer harboring an *FGFR2* gene fusion. Patient 1 did not complete ponatinib therapy due to the development of a port-associated catheter thrombosis which excluded the patient from further ponatinib therapy. Patient 3 had stable disease based on RECIST measurements and not had a decrease CA19-9 as described.

**OSU-19041: Phase II study of oral Infigratinib in adult patients with advanced or metastatic solid tumors with** ***FGFR1-3*** **gene fusions or other** ***FGFR*** **genetic alterations (NCT04233567)**.

**Description:** This open-label, single arm phase 2 basket trial was a single institution study completed at The Ohio State University James Cancer Center in Columbus, Ohio, USA. Eligible patients included the following: aged 18 years or older, histologically, or cytologically confirmed diagnosis of refractory metastatic solid tumor for whom no other standard treatment options are available, and the presence of activating genomic alterations in *FGFR* (mutations, fusions, or amplifications (> 6 copies)) by any validated CLIA-certified molecular testing (FISH, PCR or sequencing data were acceptable). This study included two patients with pancreatic cancer harboring an *FGFR2* gene fusion. Patient 2 initially received pemigatinib in another clinical trial, and later received infigratinib but had disease progression. Patient 3 developed a partial response, after previously receiving ponatinib.

## Supplementary information

Supplemental Figures and Tables

## Data Availability

The data generated in this study are available upon request from the corresponding author. Summary data that can be released are included in the article and its supplementary files. Patients tested with Foundation Medicine, Inc., were not consented for the release of raw underlying genomic sequence data. Academic researchers can gain access to the FoundationCORE data analyzed in this study by contacting the corresponding author and filling out a study review committee proposal form. Researchers and institutions will be required to execute a data transfer agreement. For further questions please reach out to Foundation Medicine, Cambridge, MA’s compliance department (compliance@foundationmedicine.com).
