# Supplementary material for: FGFR2-fusions define a clinically actionable molecular subset of pancreatic cancer
Source: NPJ Precis Oncol. 2024 Sep 17;8:207. doi: 10.1038/s41698-024-00683-x (PMC11408739; doi:10.1038/s41698-024-00683-x)
Supplement: Supplementary file 1 — Supplemental Figures and Tables [file 41698_2024_683_MOESM1_ESM.pdf]

Manuscript: *FGFR2*-fusions define a clinically actionable molecular subset of pancreas cancer

Supplementary Data Table of Contents

1. Supplementary Figure 1: Treatment durability and response to chemotherapy and FGFR kinase inhibitors in patients with metastatic PDAC and *FGFR2*-fusions.
2. Supplementary Figure 2: Radiologic scans of patient 1, 2, and 4’s best response.
3. Supplementary Figure 3: CONSORT flow diagram for clinical trial OSU-14078 (NCT02272998) for ponatinib.
4. Supplementary Figure 4: Patient 2’s RECIST scores on pemigatinib.
5. Supplementary Figure 5: CONSORT flow diagram for clinical trial OSU-19041 (NCT04233567) for infigratinib.
6. Supplementary Figure 6: Patient 2’s RECIST scores on infigratinib.
7. Supplementary Figure 7: Patient 3’s RECIST scores on ponatinib.
8. Supplementary Figure 8: Patient 3’s RECIST scores on infigratinib.
9. Supplementary Figure 9: Prevalence of *FGFR1-3* CNAs, SVs, and REs in pancreatic adenocarcinoma.
10. Supplementary Figure 10: Known gain-of-function *FGFR1-3* SVs in pancreatic adenocarcinoma.
11. Supplementary Figure 11: *FGFR1-3* short variants of unknown significance (VUS) and likely pathogenic short variants in pancreatic adenocarcinoma.
12. Supplementary Figure 12: Scheme of a Phase II, telemedicine trial with pemigatinib in unresectable or metastatic pancreatic cancer with *FGFR* genomic alterations.
13. Supplementary Table 1: Comparison of patient 1-4 demographics.
14. Supplementary Table 2: Comparison of demographics characteristics between the *FGFR1-4* rearranged and *FGFR1-4* rearrangement wildtype cohort of patients.

Supplementary Data

Supplementary Figure 1

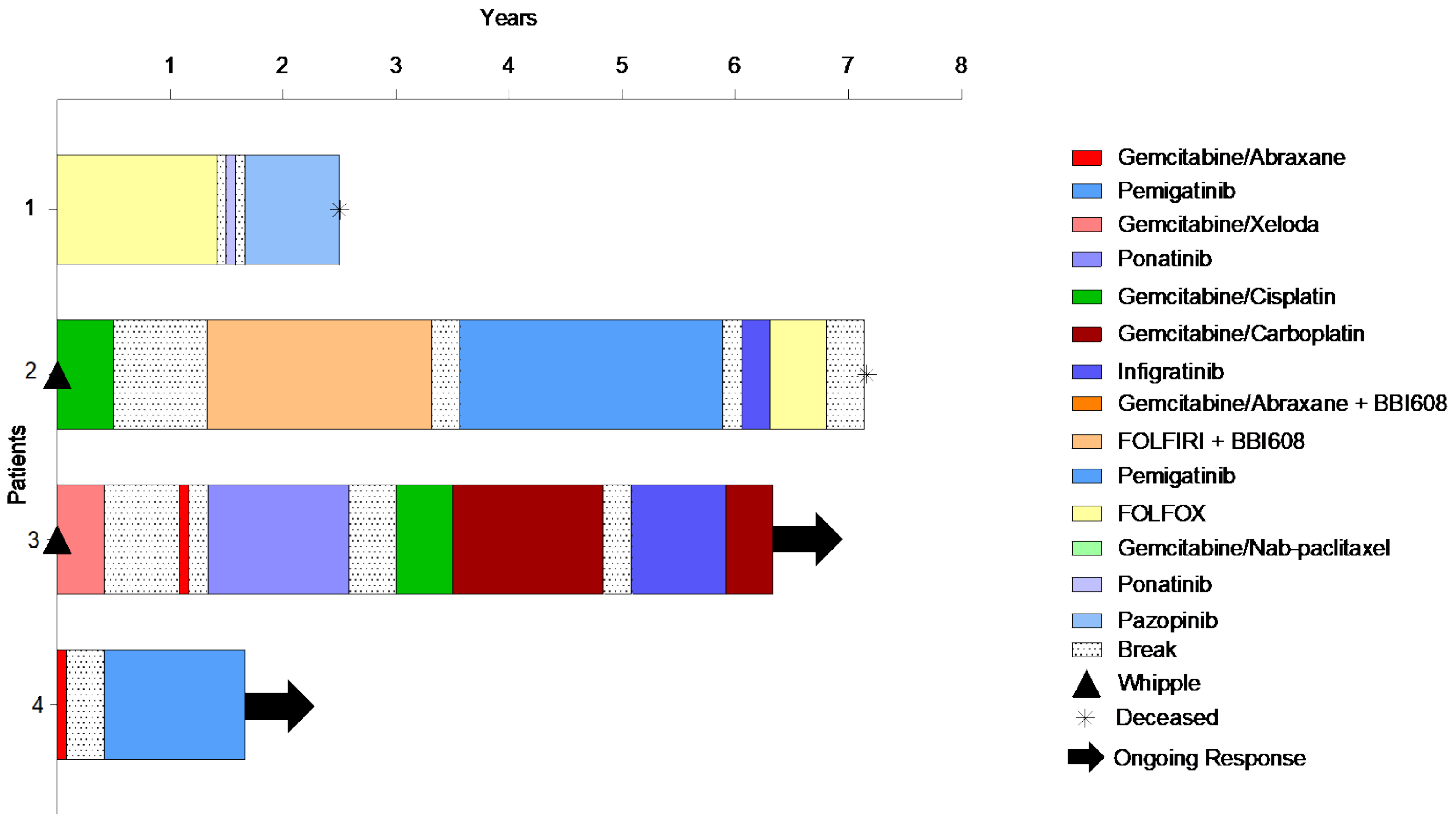

Supplementary Figure 1. Treatment durability and response to chemotherapy and FGFR kinase inhibitors in patients with metastatic PDAC and *FGFR2*-fusions. Patient 1 exhibited a best response of stable disease while on pazopanib for 10 months while patient 2 exhibited a partial response on pemigatinib for 28 months, patient 3 exhibited partial response to infigratinib for 10 months, and patient 4 exhibited an ongoing, partial response on off-label pemigatinib. Abbreviations: FOLFOX, folinic acid, fluorouracil, and oxaliplatin; BBI608, STAT3 inhibitor (Napabucasin); FOLFIRI, folinic acid, fluorouracil, irinotecan.

Supplementary Figure 2

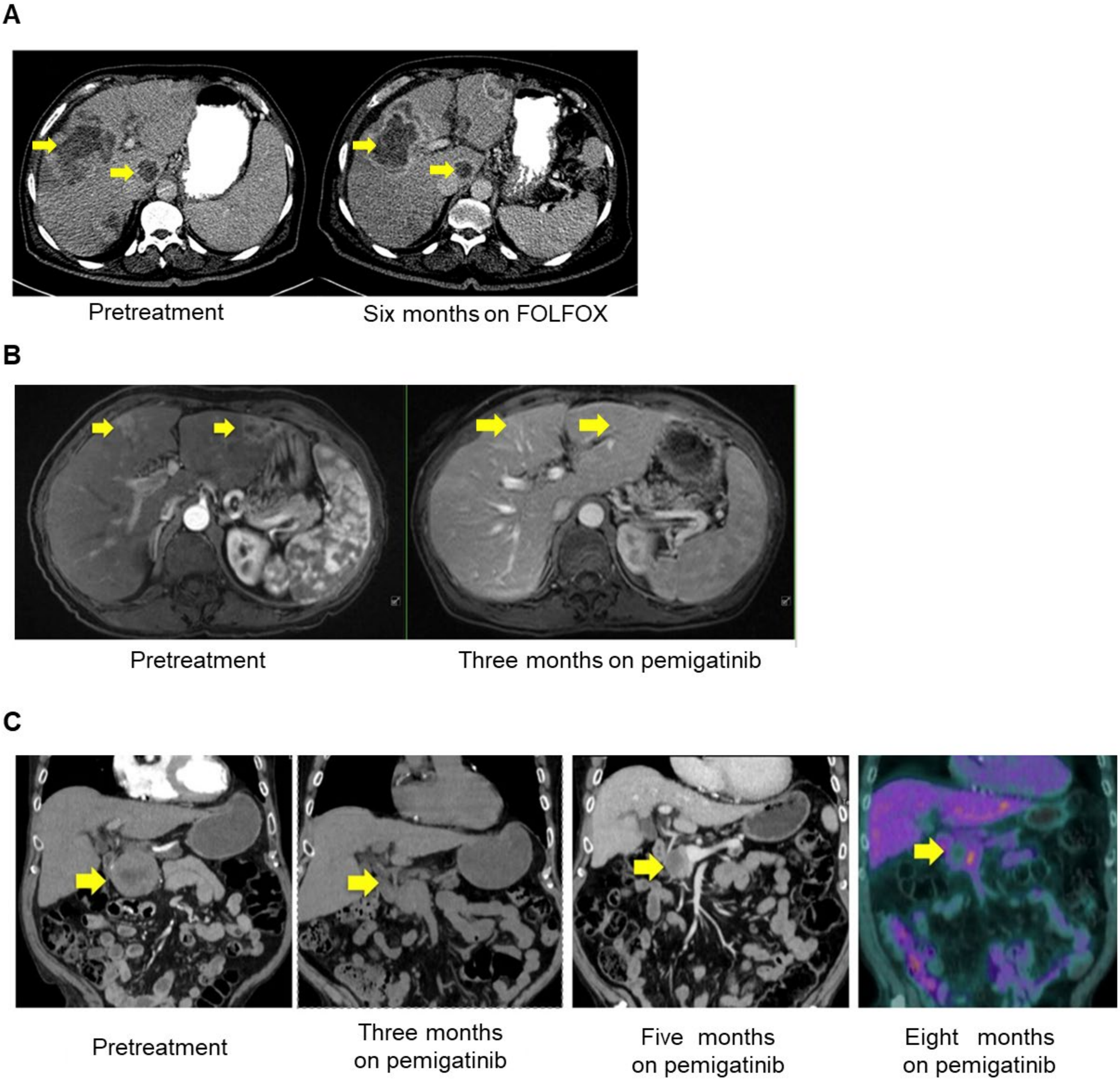

Supplementary Figure 2. Radiologic scans of patient 1, 2, and 4's best response. A, CT scans of patient 1's best response of stable disease while on FOLFOX. **B,** MRI of patient 2's best response of partial response while on pemigatinib basket trial (NCT02393248). **C,** Pretreatment, three months, and five months on pemigatinib CT scans of patient 4's partial response to off label pemigatinib. The last scan is a PET scan due to the patient's chronic kidney disease.

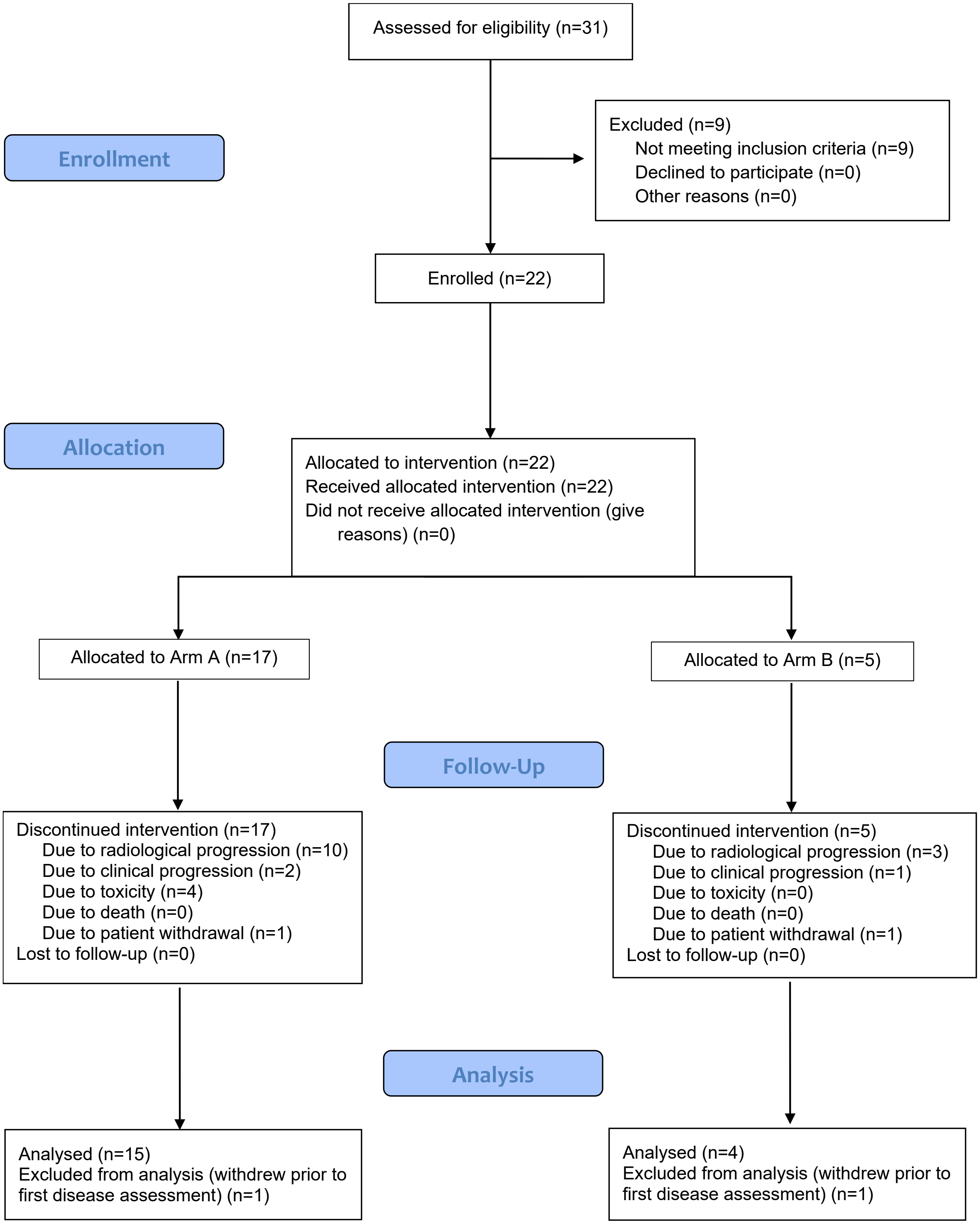

Supplementary Figure 3. CONSORT flow diagram for clinical trial OSU-14078 (NCT02272998) for ponatinib.

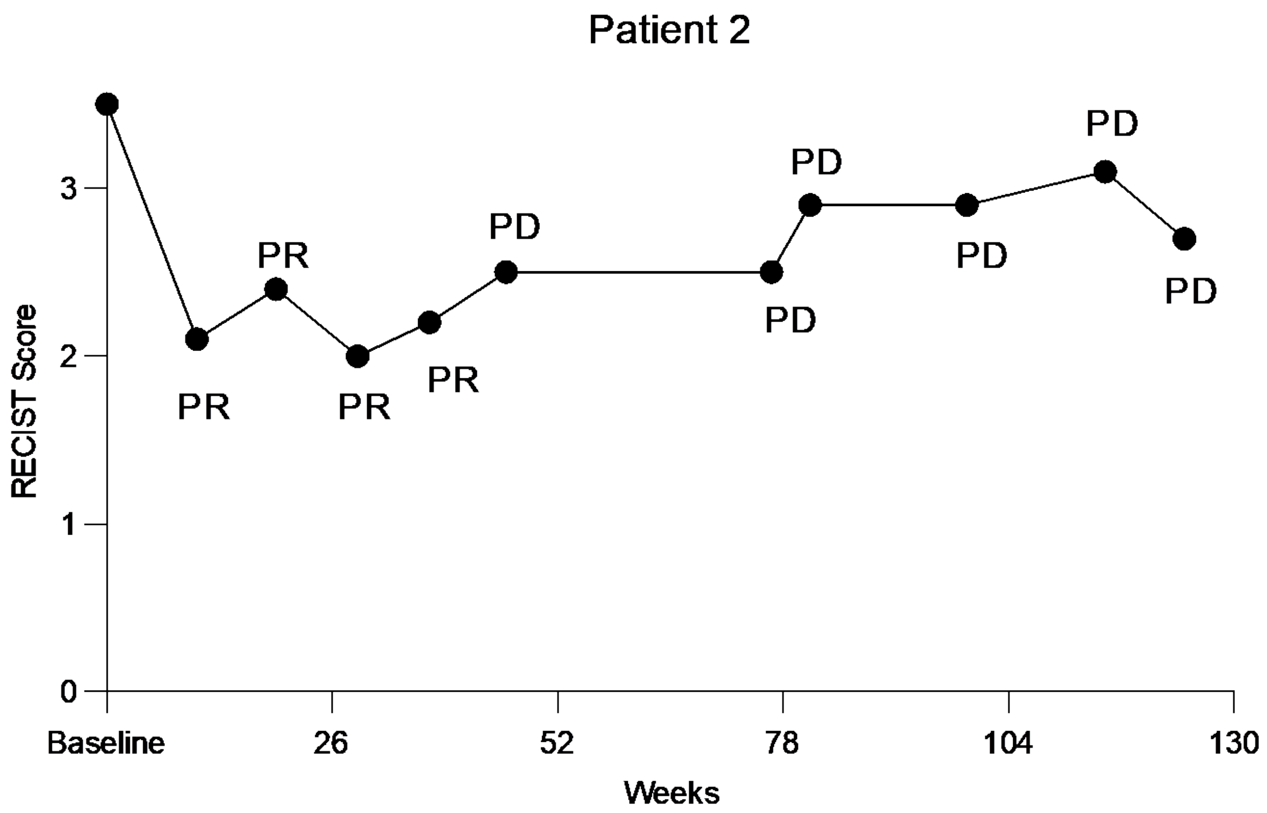

Supplementary Figure 4. Patient 2's RECIST scores on pemigatinib. Patient 2's response to pemigatinib according to RECIST scores starting with baseline scans at week 0 of infigratinib treatment. Even after disease progressed, Patient 2 still underwent treatment per Principal Investigator's request, since she was still clinically benefitting from being on therapy with no other disease evident. Abbreviations: PR, partial response; PD, progressive disease.

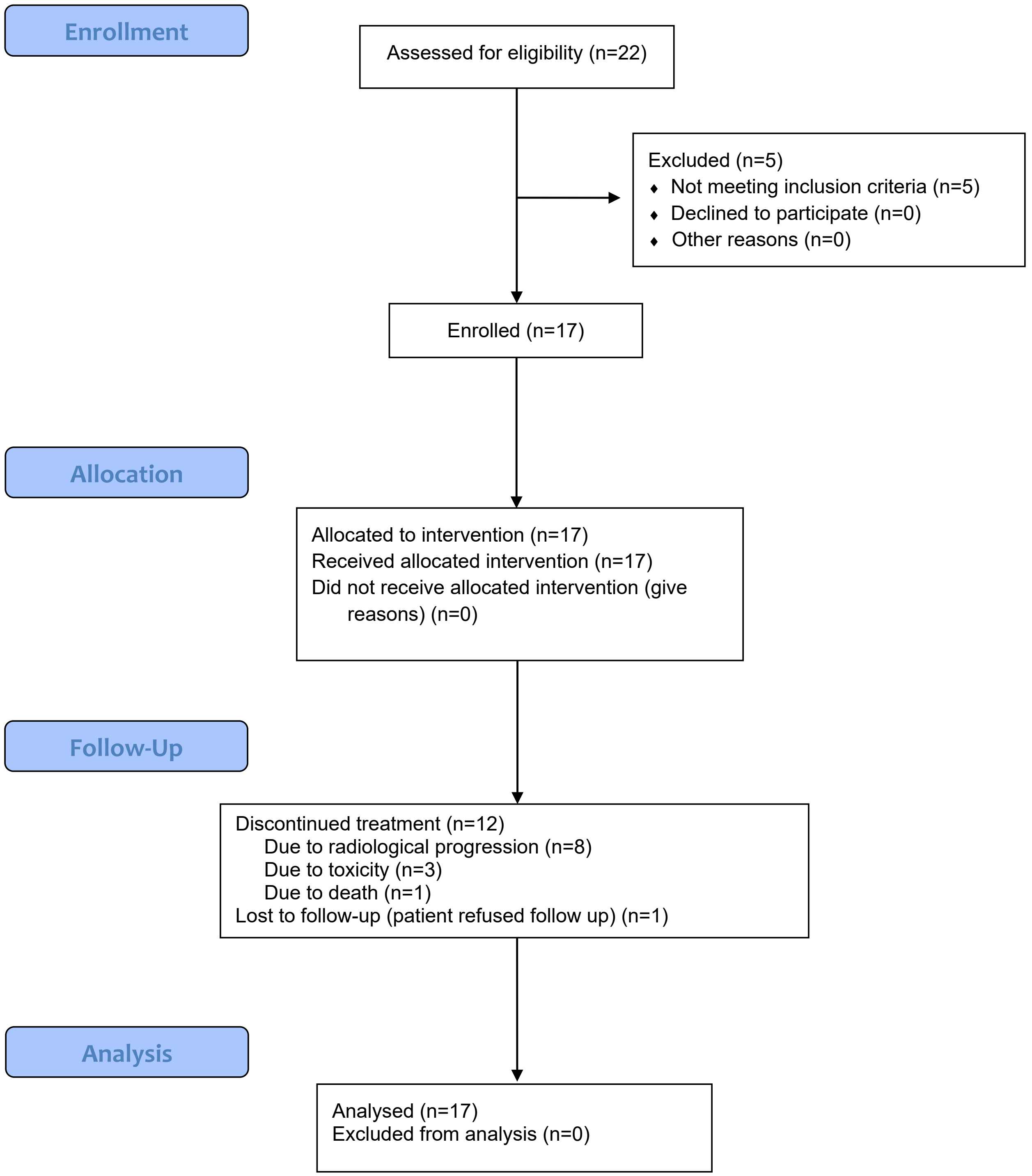

Supplementary Figure 5. CONSORT flow diagram clinical trial OSU-19041 (NCT04233567) for infigratinib.

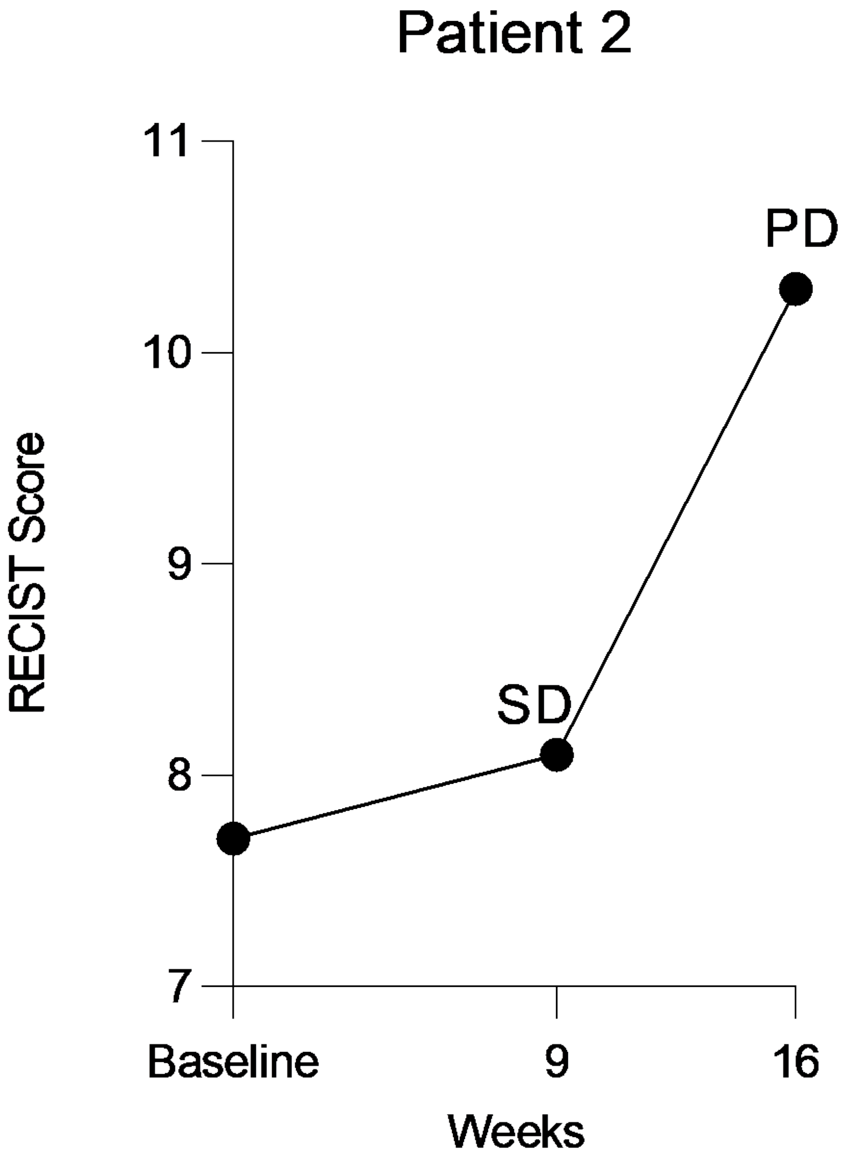

Supplementary Figure 6. Patient 2's RECIST scores on infigratinib. Patient 2's response to infigratinib according to RECIST scores starting with baseline scans at week 0 of infigratinib treatment. Abbreviations: SD, stable disease; PD, progressive disease.

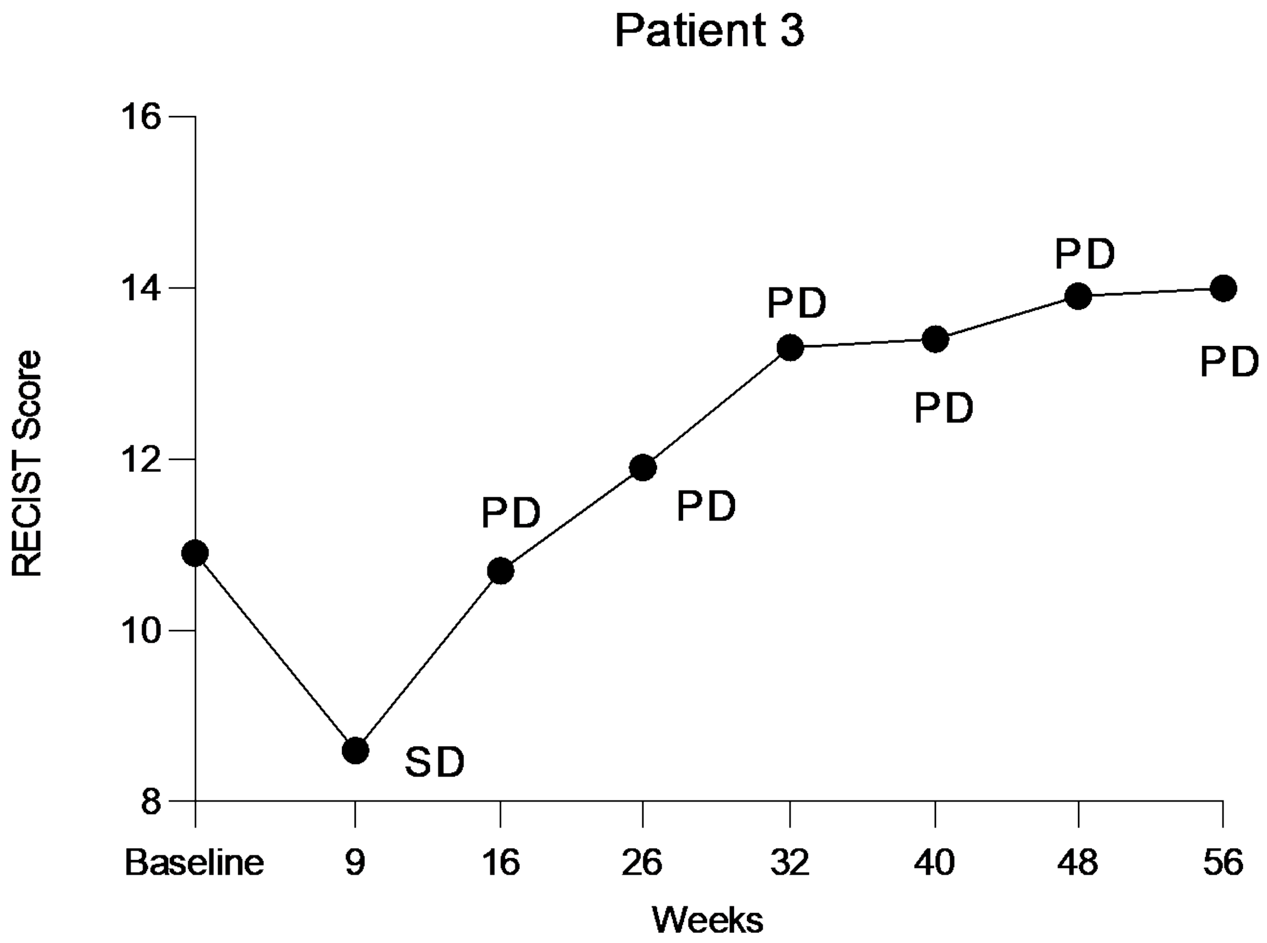

Supplementary Figure 7. Patient 3's RECIST scores on ponatinib. Patient 2's response to ponatinib according to RECIST scores starting with baseline scans at week 0 of ponatinib treatment. Treatment with ponatinib was continued after progressive disease was documented per Principal Investigator's request due to a decrease in CA19-9, clinical benefit, and disease control outside of one of the target lesions. Abbreviations: SD, stable disease; PR, partial response; PD, progressive disease.

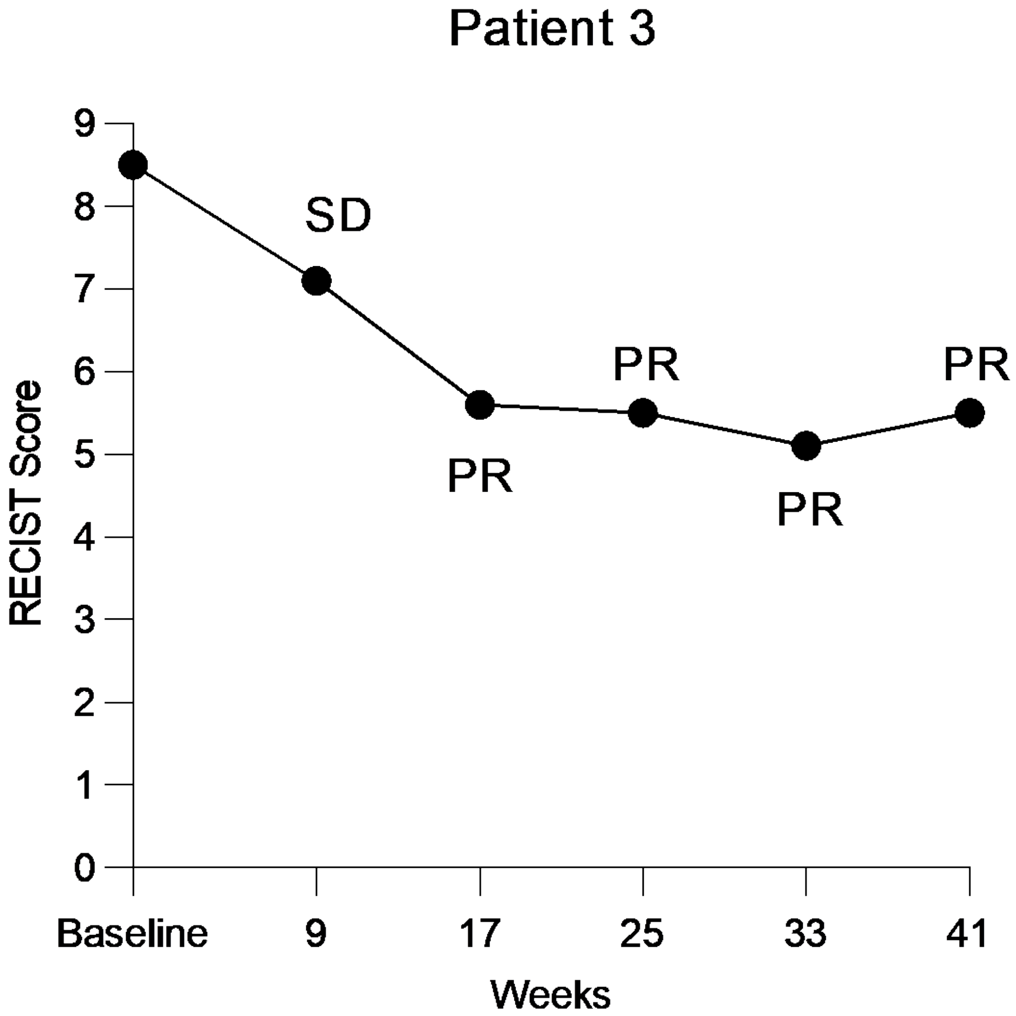

Supplementary Figure 8. Patient 3's RECIST scores on infigratinib. Patient 3's response to infigratinib according to RECIST scores starting with baseline scans at week 0 of infigratinib treatment. Abbreviations: SD, stable disease; PR, partial response; PD, progressive disease.

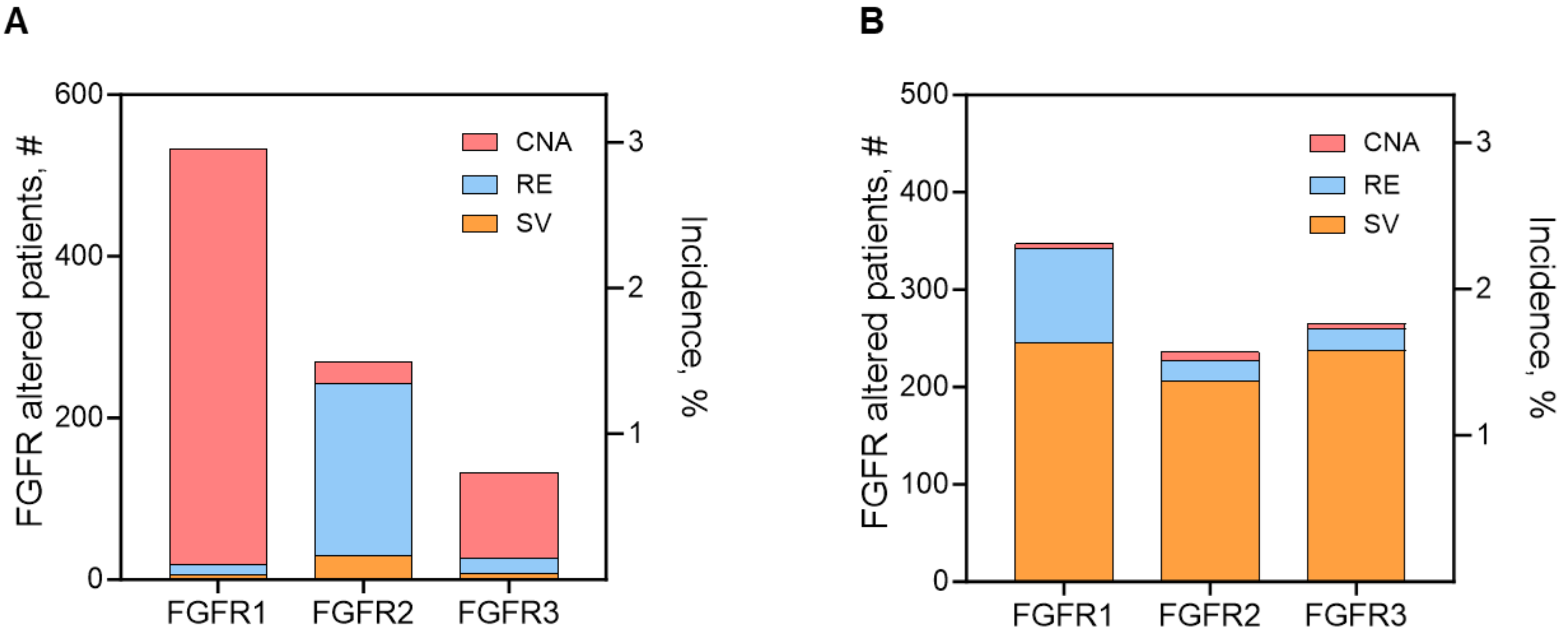

Supplementary Figure 9. Prevalence of *FGFR1-3* CNAs, SVs, and REs in pancreatic adenocarcinoma. A, *FGFR1-3* known pathogenic *FGFR1-3* alterations in pancreatic adenocarcinoma. CNAs were most observed in *FGFR1* while REs are most observed in *FGFR2*. There are very little REs and SVs with known function in *FGFR4*. **B,** *FGFR1-3* likely pathogenic and variants of unknown significance in pancreatic adenocarcinoma. REs of unknown function are most observed in *FGFR1* while SVs of unknown function occur similarly across *FGFR1-3*. This highlights the ongoing need to characterize these alterations that still have unknown function.

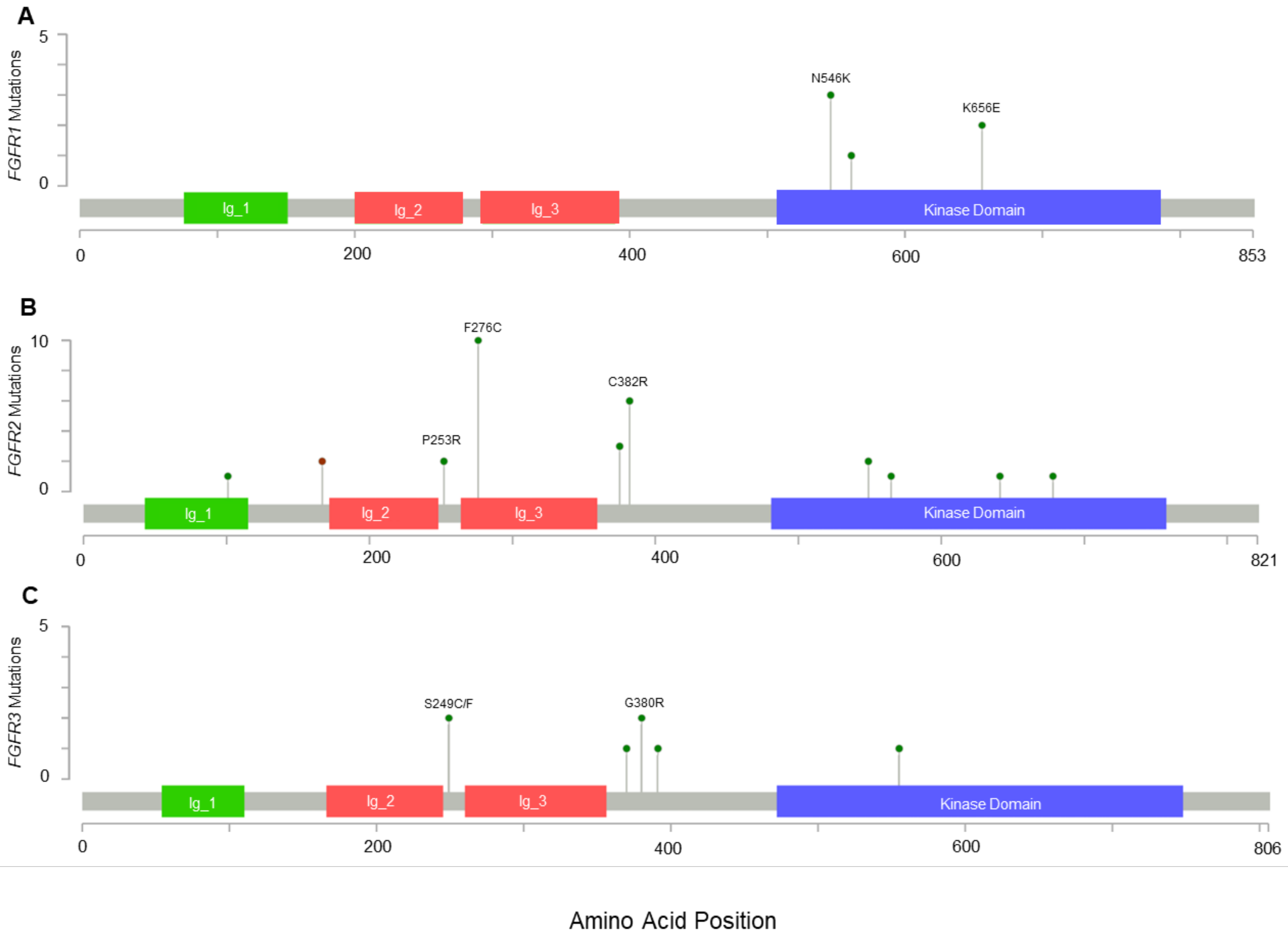

Extended Figure 10. Known gain-of-function *FGFR1-3* SVs in pancreatic adenocarcinoma. Lollipop depiction of the most common known SVs (N=18) **A,** *FGFR1* (3), **B,** *FGFR2* (10), and **C,** *FGFR3* (5) missense (green dots) alterations and in-frame indels (brown dots) observed in *FGFR*-altered pancreatic cancer. Each alteration is represented by protein domain location and frequency (height). Figure was generated using cBioPortal MutationMapper[1,2].

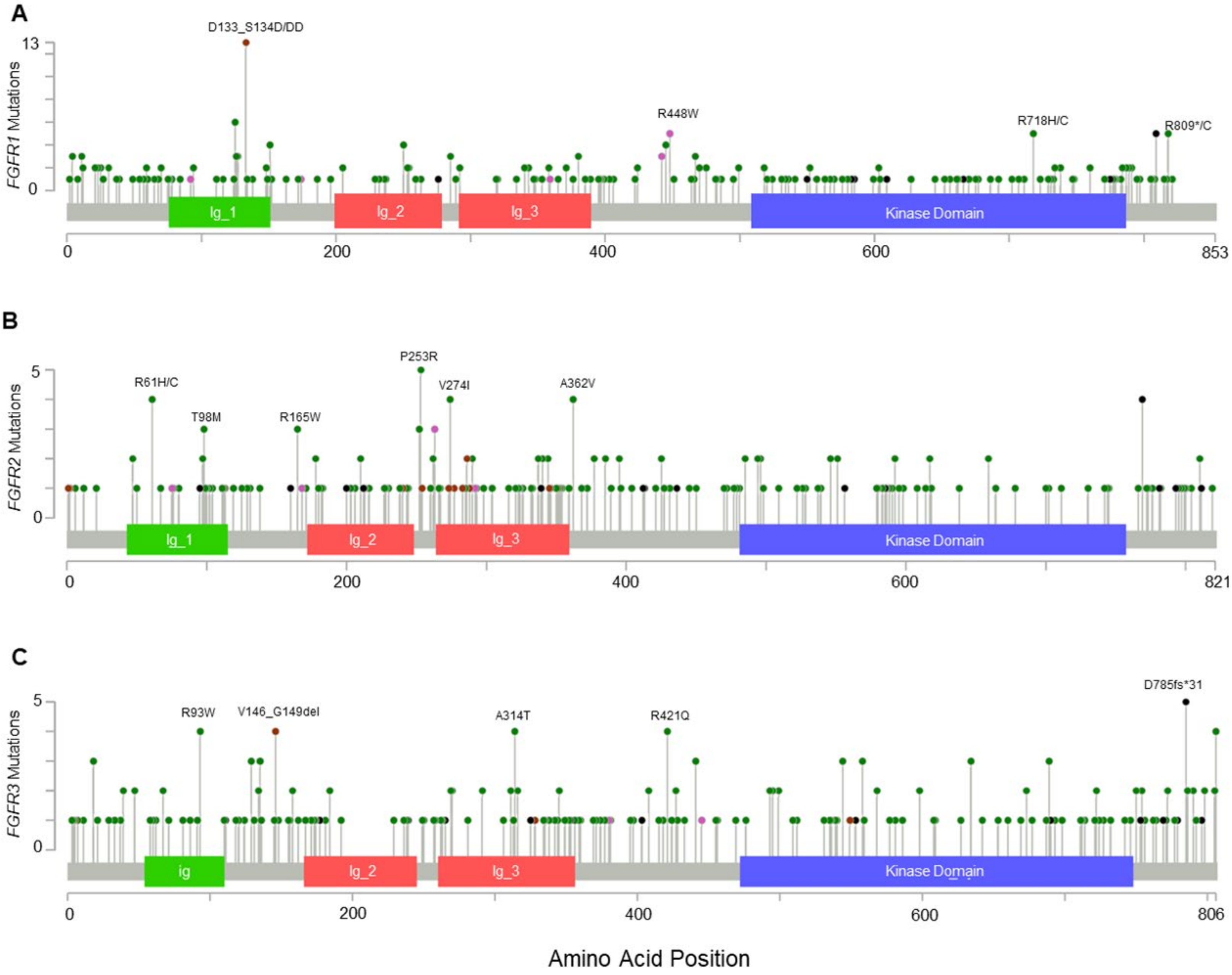

Supplementary Figure 11. ***FGFR1-3*** **short variants of unknown significance (VUS) and likely pathogenic short variants in pancreatic adenocarcinoma.** Lollipop depiction of unknown function SVs (N=563) **A,** *FGFR1* (188), **B**, *FGFR2* (183), and **C,** *FGFR3* (192) missense (green dots) alterations, truncating alterations including nonsense, nonstop, frameshift indels, and splice sites (black dots), and in-frame indels (brown dots), and all other alterations (pink dots) observed in *FGFR*-altered pancreatic cancer. Some of the likely pathogenic *FGFR* SVs include *FGFR1 S125L, FGFR1 R445W, FGFR1 G818R, FGFR2 P253R, FGFR2 Y769*, FGFR2 M537I, FGFR2 372C,* and *FGFR3 R621H.* These SVs labeled as likely pathogenic are rare *FGFR* mutations that warrant further investigation. Each alteration is represented by protein domain location and frequency (height). Figure was generated using cBioPortal MutationMapper[1,2].

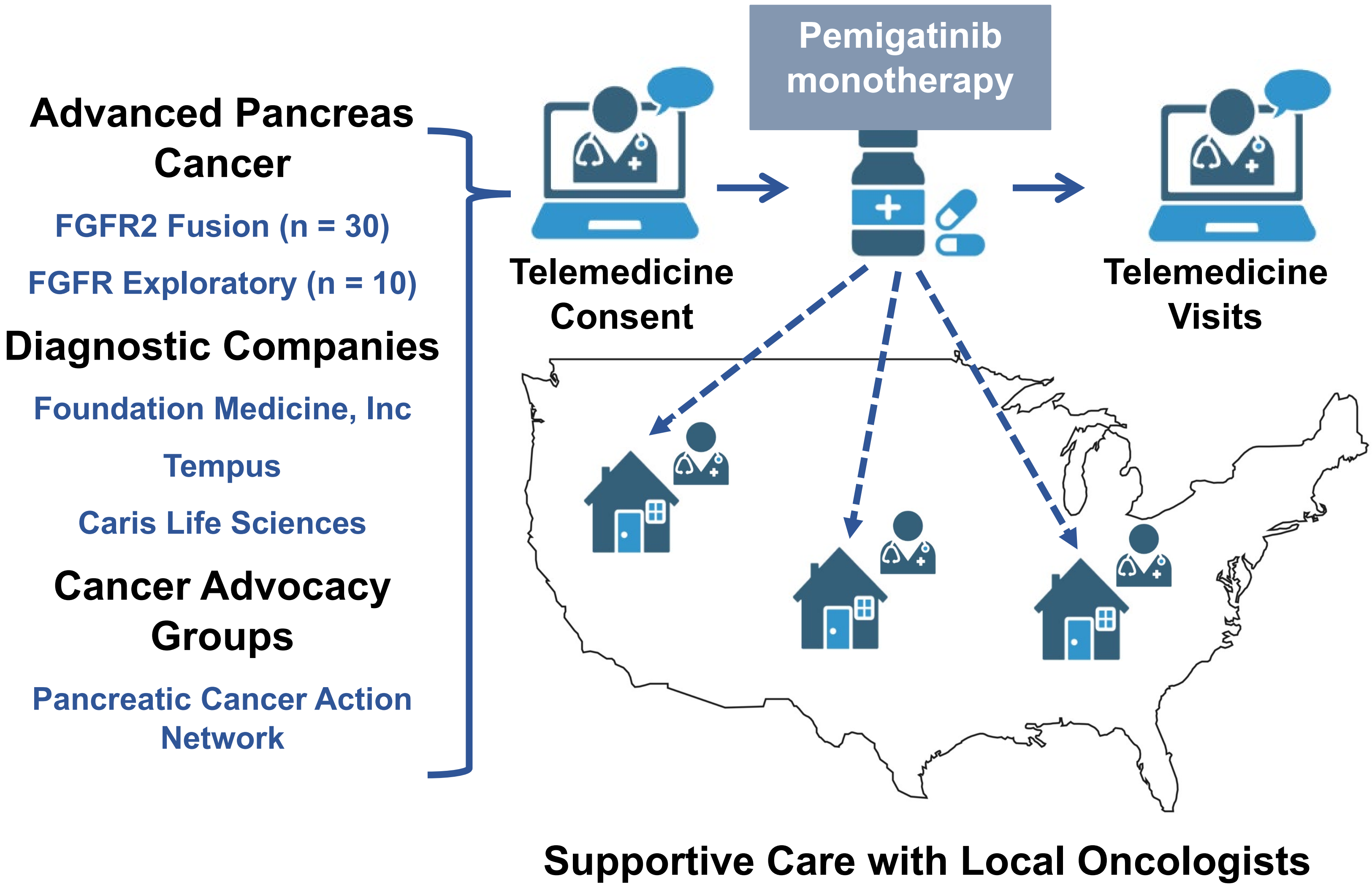

Supplementary Figure 12. Scheme of a Phase II, telemedicine trial with pemigatinib in unresectable or metastatic pancreatic cancer with *FGFR* genomic alterations.

Supplementary Table 1

Variable	Patient 1	Patient 2	Patient 3	Patient 4
Age (at diagnosis)	61	44	71	79
Gender	Male	Female	Male	Male
Race	African American	White	White	White
Cancer Histology	Poorly differentiated carcinoma	Moderately differentiated adenocarcinoma	Adenocarcinoma	Adenocarcinoma
# of systemic therapies	4	7	6	2
Systemic therapies	Gemcitabine/nab-paclitaxel; FOLFOX; Ponatinib; Pazopanib	Gemcitabine/Cisplatin; Gemcitabine/nab-paclitaxel/BB1608; FOLFIRI/BB1608; Pemigatinib; Infigratinib; FOLFIRINOX	Gemcitabine/Capecitabine; Gemcitabine/nab-paclitaxel; Ponatinib; Gemcitabine/Cisplatin; Gemcitabine/Carboplatin; Infigratinib	Gemcitabine/nab-paclitaxel; Pemigatinib
Genomic alteration	FGFR2-INA; FGFR2 amplification	FGFR2-USP33	FGFR2-CEP55	FGFR2-INA; FGFR2 amplification
Smoking status (pack years)	21	0	0	15

Supplementary Table 2

Supplementary Table 1. Comparison of Patient 1-4 demographics.

		FGFR1-4 RE mt (N=245)	*FGFR1-4 RE* wt (N=29984)
Gender: Males (n, %)[1]		116 (47.3)	15995 (53.3)
Median age (IQR; years)[2]		61 [52-69]	66 [59-73]
Age ≥ 18 years (n, %)		244 (99.6)	29943 (99.9)
Genomic ancestry (n, %)[3]	AFR	27 (11.0)	2831 (9.4)
	AMR	15 (6.1)	1444 (4.8)
	EAS	11 (4.5)	1024 (3.4)
	EUR	184 (75.1)	23526 (78.5)
	SAS	2 (0.8)	229 (0.8)
Metastatic (n, %)[4]		204 (78.4)	14849 (49.1)

Supplementary Table 2. Comparison of demographics characteristics between the *FGFR1-4* rearranged and *FGFR1-4* rearrangement wildtype cohort of patients. Gender variable had a missingness rate of 0.03% (n=10). Age variable had a missingness rate of 0.08% (n=25) in the dataset. Median age comparison was significant by Wilcoxon rank sum test, P=2.3e-10. Genomic ancestry variable had a missingness rate of 3.1% (n=936). Metastatic variable had a missingness rate of 8.1% (n=2457) in the dataset. Abbreviations: AFR, Africa; AMR, Americas; EAS, East-Asia; EUR, Europe; SAS, South-Asia

References

1. Cerami, E., *et al.* The cBio cancer genomics portal: an open platform for exploring multidimensional cancer genomics data. *Cancer Discov* **2**, 401-404 (2012).
2. Gao, J., *et al.* Integrative analysis of complex cancer genomics and clinical profiles using the cBioPortal. *Sci Signal* **6**, pl1 (2013).
